# National Surveillance for Acute Flaccid Myelitis — United States, 2018–2020

**DOI:** 10.15585/mmwr.mm7044a2

**Published:** 2021-11-05

**Authors:** Sarah Kidd, Eileen Yee, Randall English, Shannon Rogers, Brian Emery, Halle Getachew, Janell A. Routh, Adriana S. Lopez

**Affiliations:** ^1^Division of Viral Diseases, National Center for Immunization and Respiratory Diseases, CDC; ^2^Cherokee Nation Assurance, Arlington, Virginia; ^3^Strategic Innovative Solutions, Clearwater, Florida.

Acute flaccid myelitis (AFM), a recognized complication of certain viral infections, is a serious neurologic condition that predominantly affects previously healthy children and can progress rapidly, leading to respiratory insufficiency and permanent paralysis. After national AFM surveillance began in 2014, peaks in AFM cases were observed in the United States in 2014, 2016, and 2018 (*1*). On the basis of this biennial pattern, an increase in AFM was anticipated in 2020. To describe the epidemiology of confirmed AFM cases since 2018, demographic, clinical, and laboratory information collected as part of national AFM surveillance was reviewed. In 2018, a total of 238 confirmed AFM cases were reported to CDC, compared with 47 cases in 2019 and 32 in 2020. Enterovirus D68 (EV-D68) was detected in specimens from 37 cases reported in 2018, one case in 2019 and none in 2020. Compared with 2018, cases reported during 2019–2020 occurred in older children and were less frequently associated with upper limb involvement, febrile or respiratory prodromal illness, or cerebrospinal fluid (CSF) pleocytosis. These findings suggest that the etiologies of AFM in 2019 and 2020 differed from those in 2018. The absence of an increase in cases in 2020 reflects a deviation from the previously observed biennial pattern, and it is unclear when the next increase in AFM should be expected. Clinicians should continue to maintain vigilance and suspect AFM in any child with acute flaccid limb weakness, particularly in the setting of recent febrile or respiratory illness.

Similar to poliomyelitis caused by poliovirus (an enterovirus), AFM is characterized by sudden onset of limb weakness and lesions in the gray matter of the spinal cord. CDC began conducting national surveillance for AFM in 2014 after a cluster of cases of acute flaccid limb weakness among previously healthy children who had no laboratory or epidemiologic evidence of poliovirus infection was reported in Colorado (*2*). Since then, national surveillance has demonstrated biennial peaks in AFM cases during the late summer and early fall in 2014, 2016, and 2018 (*1*).

AFM is an unusual but known complication of certain viral infections, including those from West Nile virus and nonpolio enteroviruses (*3*,*4*). Pathogens are rarely isolated from the CSF of AFM patients (*2*,*5*). However, enteroviruses (EVs) are the most common pathogens detected from AFM patient respiratory and stool specimens; EV-D68 is the most common enterovirus type detected, and additional laboratory and animal model data suggest that EV-D68 is the primary driver of increases in AFM during peak years (*2*,*5*–*7*). Previously published AFM surveillance data through 2018 suggest that case characteristics and etiology differ during peak versus nonpeak years (*8*). CDC examined national surveillance data to further understand the epidemiology and etiology of AFM and describe trends since 2018, the most recent peak year.

As part of national surveillance, health departments report cases meeting the clinical criterion for AFM (acute flaccid limb weakness) to CDC via a patient summary form that includes demographic and clinical information. Health departments also send CDC important elements from the patient’s medical record, and data from these records are abstracted using a standardized worksheet. In addition, health departments and clinicians submit available CSF, respiratory, serum, and stool specimens to CDC. Testing protocols at CDC include enterovirus/rhinovirus (EV/RV)* testing using methods that have been described previously (*2*).

Patient summary form, chart abstraction, and laboratory data were analyzed to describe trends in confirmed AFM cases since surveillance began in August 2014 and to describe case characteristics in 2018, 2019, and 2020. Confirmed AFM was defined as acute onset of flaccid limb weakness accompanied by magnetic resonance imaging demonstrating a spinal cord lesion largely restricted to gray matter and spanning one or more vertebral segments (*9*). Reported EV/RV results include external laboratory results that were documented in the available medical records and CDC laboratory results. This activity was reviewed by CDC and was conducted consistent with applicable federal law and CDC policy.^†^

A total of 238 confirmed cases were reported to CDC in 2018, 47 cases were reported in 2019, and 32 cases were reported in 2020 (Figure). During each year, at least 90% of cases (94% in 2018 and 91% in 2019 and 2020) occurred among children aged <18. Compared with cases in the most recent peak year (2018), AFM patients in 2019 and 2020 were older (median age = 6.6 and 9.2 years, respectively, versus 5.3 years in 2018) and were less frequently associated with upper limb involvement (74% and 59%, versus 84%), prodromal respiratory or febrile illness (57% and 63%, versus 92%), or CSF pleocytosis (49% and 48%, versus 87%) (Table 1). Lower limb involvement was more common among patients with AFM reported in 2019 and 2020 than among 2018 cases (74% and 81%, versus 44%).

**FIGURE:**
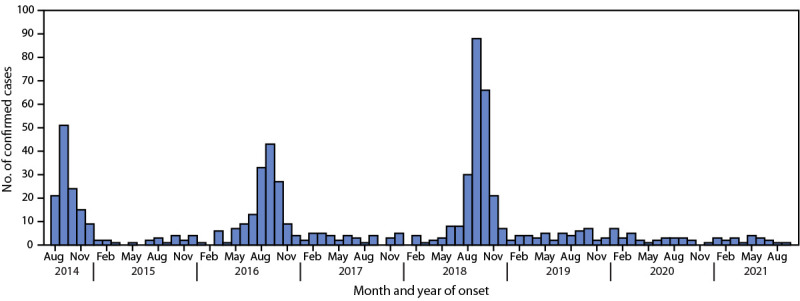
Confirmed cases of acute flaccid myelitis (N = 670), by month of onset — United States, August 2014–September 2021* * As of October 23, 2021.

**TABLE 1:** Demographic and clinical characteristics of patients with confirmed acute flaccid myelitis — United States, 2018–2020

Characteristic	No. (%)		
	2018 (N = 238)	2019 (N = 47)	2020 (N = 32)
**Median age, yrs (IQR)**	5.3 (3.3–8.2)	6.6 (2.9–12.8)	9.2 (3.5–14.5)
**Sex**			
Male	138 (58)	15 (32)	16 (50)
Female	100 (42)	32 (68)	16 (50)
Race/Ethnicity*			
Asian	8 (3)	2 (4)	3 (9)
Black or African American	21 (9)	7 (15)	4 (13)
Hispanic	47 (20)	12 (26)	8 (25)
Native Hawaiian or Other Pacific Islander	1 (0.4)	0 (—)	0 (—)
White	125 (53)	18 (38)	12 (38)
Multiracial	4 (2)	1 (2)	0 (—)
Unknown	32 (13)	7 (15)	5 (16)
**Geographic region**			
West	56 (24)	19 (40)	8 (25)
Midwest	61 (26)	7 (15)	7 (22)
South	80 (34)	18 (38)	11 (34)
Northeast	41 (17)	3 (6)	6 (19)
**Limbs affected**			
Upper	199 (84)	35 (74)	19 (59)
Lower	132 (55)	35 (74)	26 (81)
**Illness in the 4 weeks before onset of limb weakness**			
Any illness	223 (94)	32 (68)	20 (63)
Respiratory illness	187 (79)	23 (49)	14 (44)
Fever	174 (73)	14 (30)	12 (38)
Respiratory illness or fever	218 (92)	27 (57)	20 (63)
Gastrointestinal illness	80 (34)	12 (26)	3 (9)
**Timing of preceding illness, median days before limb weakness^†^ (IQR)**			
Any illness	5 (3–8)	4 (3–7)	5 (2–13)
Respiratory illness	5 (3–8)	5 (3–14)	5.5 (2.5–13.5)
Fever	3 (1–5)	3 (2–5)	2.5 (1–5.5)
Respiratory illness or fever	5 (3–7)	4 (3–6)	5 (2–13)
Gastrointestinal illness	2 (1–6)	3.5 (2–6)	4 (0–14)
**CSF microscopic examination, no./total no. (%)**			
CSF pleocytosis	183/210 (87)	21/43 (49)	13/27 (48)
Median white blood cell count, cells/mm^3^ (IQR)^§^	94 (43–163)	107 (44–182)	36 (9–55)
**Characteristics of hospitalization and clinical care**			
Hospitalized	233 (98)	46 (98)	32 (100)
**Timing of hospitalization in relationship to onset of weakness among those hospitalized, no./total no. (%)**			
Before onset of limb weakness	25/233 (11)	6/46 (13)	1/32 (3)
After onset of limb weakness	206/233 (88)	40/46 (87)	31/32 (97)
Unknown if hospitalized before or after onset of limb weakness	2/233 (1)	0/46 (—)	0/32 (—)
**Days from onset of weakness to hospitalization (among those hospitalized after onset of weakness), no./total no. (%)**			
Median (IQR)	1 (0–2)	1 (0–1)	1 (1–1)
0–1	134/206 (65)	33/40 (83)	26/31 (84)
2–3	52/206 (25)	5/40 (13)	4/31 (13)
4–7	10/206 (5)	2/40 (5)	0/31 (—)
>7	10/206 (5)	0/40 (—)	1/31 (3)
**Treatment**			
Steroids, no IVIG	55 (23)	14 (30)	7 (22)
IVIG, no steroids	54 (23)	12 (26)	6 (19)
Both steroids and IVIG	81 (34)	15 (32)	14 (44)
Plasma exchange	32 (13)	10 (21)	10 (31)
Admitted to ICU	129 (54)	24 (51)	19 (59)
Respiratory support	65 (27)	16 (34)	6 (19)
Mechanical ventilation	55 (23)	13 (28)	5 (16)
**Location of first medical encounter after onset of weakness**			
Emergency department	134 (56)	32 (68)	24 (75)
Primary care provider	49 (21)	4 (9)	3 (9)
Urgent care provider	16 (7)	4 (9)	1 (3)
Had onset of weakness during an inpatient hospitalization	25 (11)	6 (13)	1 (3)
Unknown or other	14 (6)	1 (2)	3 (9)
**Days from onset of weakness to first medical encounter (excluding those hospitalized before onset of weakness), no./total no. (%)**			
Median (IQR)	0 (0–1)	0 (0–1)	0 (0–0)
0–1	160/213 (75)	36/41 (88)	30 /31 (97)
2–3	34/213 (16)	3/41 (7)	0/31 (—)
4–7	4/213 (2)	0/41 (—)	0/31 (—)
>7	2/213 (1)	2/41 (5)	0/31 (—)
Unknown	13/213 (6)	0/41 (—)	1/31 (3)

**Abbreviations:** CSF = cerebrospinal fluid; ICU = intensive care unit; IQR = interquartile range; IVIG = intravenous immunoglobulin.

* Persons reported in the following groups are non-Hispanic: Asian, Black or African American, Native Hawaiian or Other Pacific Islander, and White.

^†^ Timing calculated among cases with the prodromal illness/symptom and documented valid dates of onset.

^§^ Median cells/mm^3^ was calculated among cases with CSF pleocytosis (>5 white blood cells/mm^3^).

Almost all (98%–100%, depending on year) patients with confirmed AFM reported during 2018–2020 were hospitalized, 51%–59% were admitted to an intensive care unit, and 16%–28% required intubation and mechanical ventilation. During each year, the emergency department was the most common location of the first medical encounter after onset of weakness. During 2018–2020, an increasing proportion of patients with confirmed AFM sought medical care and were hospitalized within 1 day of weakness onset. The percentage of patients seeking medical care within 1 day of weakness onset increased from 75% in 2018 to 97% in 2020, and the percentage of patients hospitalized within 1 day increased from 65% to 84%.

In 2018, among patients with confirmed AFM who were tested, EV/RV was detected in 50%, and EV-D68 was the most common EV/RV type detected (Table 2). In contrast, although a similar proportion of AFM patients were tested for EV/RV each year, EV/RV was detected in only 37% of 2019 cases and in 26% of 2020 cases. EV-D68 was detected in 37 cases in 2018, compared with a single case in 2019 and no cases in 2020. During each year, the highest yield of EV/RV detection was among respiratory specimens.

**TABLE 2:** Enterovirus/rhinovirus results from respiratory, stool, cerebrospinal fluid, and serum specimens collected from patients with confirmed acute flaccid myelitis — United States, 2018*–2020

Specimen source	No. (%)		
	2018 (N = 238)	2019 (N = 47)	2020 (N = 32)
**Any source^†^**			
All patients with results	223 (94)	43 (91)	31 (97)
Patients with positive results	112 (50)	16 (37)	8 (26)
EV/RV type results^§^			
EV-D68	37	1	0
EV-A71	13	2	1
Rhinoviruses	10	1	3
Other typed enteroviruses	8	2	0
Unknown or not typed	46	10	4
**Respiratory^†^**			
All patients with results	194 (82)	39 (83)	27 (84)
Patients with positive results	97 (50)	13 (33)	7 (26)
EV/RV type results^§^			
EV-D68	37	1	0
EV-A71	11	0	0
Rhinoviruses	10	1	3
Other typed enteroviruses	1	0	0
Unknown or not typed	40	11	4
**Stool**			
All patients with results	111 (47)	23 (49)	15 (47)
Patients with positive results	25 (23)	6 (26)	2 (13)
EV/RV type results^§^			
EV-D68	3	0	0
EV-A71	2	2	1
Rhinoviruses	0	0	0
Other typed enteroviruses	7	2	0
Unknown or not typed	13	2	1
**Cerebrospinal fluid**			
All patients with results	191 (80)	39 (83)	29 (91)
Patients with positive results	9 (5)	0 (—)	0 (—)
EV/RV type results^§^			
EV-D68	2	0	0
EV-A71	1	0	0
Rhinoviruses	0	0	0
Other typed enteroviruses	0	0	0
Unknown or not typed	6	0	0
**Serum**			
All patients with results	108 (45)	29 (62)	21 (66)
Patients with positive results	4 (4)	0 (—)	1 (5)
EV/RV type results^§^			
EV-D68	1	0	0
EV-A71	0	0	1
Rhinoviruses	0	0	0
Other typed enteroviruses	2	0	0
Unknown or not typed	1	0	0

**Abbreviations:** EV = enterovirus; RV = rhinovirus.

* This table includes updated laboratory information and supersedes previously published data on the 2018 cases.

^†^ Some patients had multiple positive specimens. In addition, respiratory coinfection with two EV/RV types was detected in two cases in 2018 (EV-D68 and echovirus 6 in one case, and EV-D68 and RV-A2 in another case).

^§^ Percentage not calculated.

## Discussion

In a departure from the previously observed pattern of biennial peaks in AFM cases in 2014, 2016, and 2018, there was no increase in the number of reported AFM cases in 2020. The number of confirmed AFM cases during 2019–2020 remained low and was consistent with previous nonpeak years. In addition, 2019–2020 cases differed from 2018 cases: patients were older; more likely to have lower limb involvement; and less likely to have upper limb involvement, prodromal illness, CSF pleocytosis, or specimens that tested positive for EV-D68. Upper limb involvement, prodromal respiratory illness, and CSF pleocytosis were characteristic features of 2018 cases. These findings are consistent with an earlier report of differences between AFM case characteristics during peak and nonpeak years (*8*) and likely reflect differences in AFM etiology. Specifically, increases in AFM during peak years since 2014 appear to be mostly associated with EV-D68, whereas AFM during nonpeak years likely represents a mixture of etiologies.

Regardless of etiology, AFM can progress rapidly and lead to respiratory insufficiency that requires intubation and mechanical ventilation. Persons with signs and symptoms of AFM should be immediately hospitalized and their respiratory status monitored. Although there is no proven treatment for AFM, hospitalization facilitates patient evaluation, diagnosis or exclusion of other neurologic conditions, and appropriate medical management. Notably, during 2018–2020, the proportion of patients that were hospitalized within 1 day increased. It is possible that certain features of 2019–2020 cases (e.g., older age or lower limb predominance) facilitated earlier recognition of the signs and symptoms of neurologic weakness, although this trend might also reflect increased public and clinician awareness of AFM since 2018.

The findings in this report are subject to at least two limitations. First, this analysis was based on AFM cases reported to CDC and might underestimate the actual number of AFM cases in the United States. Second, clinical information was obtained from the patient summary form, which was completed by the health department, or from the medical records, which could be incomplete. Similarly, laboratory data were limited to results documented in the medical records shared with CDC or specimens tested at CDC.

It is not entirely clear why AFM cases did not increase in 2020. Nonpharmaceutical interventions implemented during the COVID-19 pandemic (e.g., face masks, physical distancing, and reduced in-person school attendance) might have reduced transmission of EV-D68 and other enteroviruses associated with AFM. EV-D68 is a respiratory enterovirus, and other respiratory viruses such as influenza and respiratory syncytial virus (RSV) were noted to have decreased circulation during 2020 (*10*). As a group, EV/RV circulation was also attenuated, although to a lesser degree than influenza or RSV (*10*). It is also unclear when the next increase in AFM should be expected. AFM should be suspected in any child with acute flaccid limb weakness, especially among those with a recent history of a febrile or respiratory illness. Clinicians should remain vigilant for this condition in 2021 and report potential cases to their public health department.

**Summary**

**What is already known about this topic?**

Biennial peaks in reported acute flaccid myelitis (AFM) cases occurred in late summer and early fall in 2014, 2016, and 2018.

**What is added by this report?**

The number of AFM cases in 2019 and in 2020 was consistent with previous nonpeak years. Compared with 2018, cases reported during 2019–2020 were more likely to have lower limb involvement and less likely to have prodromal illness, upper limb involvement, cerebrospinal fluid pleocytosis, or detection of enterovirus D68.

**What are the implications for public health practice?**

It is unclear when another increase in AFM will occur. Clinicians should maintain vigilance and suspect AFM in any child with acute flaccid limb weakness, particularly following a recent febrile or respiratory illness.

## Footnotes

* EVs and RVs are closely related picornaviruses. Most available reverse transcription–polymerase chain reaction tests for EV amplify a viral gene sequence that is highly conserved between EVs and RVs. Therefore, these tests do not distinguish between EVs and RVs and additional testing is needed to identify the specific virus that has been detected.
